# Merkel cell carcinoma coexisting with squamous cell carcinoma^⋆^^☆^

**DOI:** 10.1016/j.abd.2024.10.006

**Published:** 2025-04-07

**Authors:** Mariana Abdo de Almeida, Neusa Yuriko Sakai Valente, Mariangela Cristina Crispino Barata, Emílio Martins Curcelli, Bruna Nascimento Arruda Scabello

**Affiliations:** a Department of Pathological Anatomy, Instituto de Assistência Médica ao Servidor Público Estadual, São Paulo, SP, Brazil; b Department of Dermatology, Hospital do Servidor Público Estadual de São Paulo, São Paulo, SP, Brazil

Dear Editor,

Merkel cell carcinoma (MCC) is a rare neuroendocrine carcinoma, the origin of which is still debated.^1^ It is a highly aggressive neoplasm, with a predilection for sun-exposed areas, mainly the head and neck, most commonly affecting elderly patients with no gender predilection.^2^

Due to its rarity, association with other skin neoplasms is possible, the most commonly described being the association with squamous cell carcinoma (SCC).^3^, ^4^

A 78-year-old female patient reported the appearance of a lesion on her right upper limb approximately four months before, with progressive growth. She denied other previous neoplastic lesions.

On physical examination, the lesion had an erythematous base, was slightly infiltrative, presented an hematic crust and keratotic surface, and showed atypical vessels on dermoscopy.

An excisional biopsy of the lesion was performed, with the following diagnostic hypotheses: basal cell carcinoma, amelanotic melanoma, or Merkel cell carcinoma. Histopathology and immunohistochemistry showed Merkel cell carcinoma associated with moderately differentiated and invasive squamous cell carcinoma, with free surgical margins (Fig. 1, Fig. 2). There was positivity for neuroendocrine markers such as synaptophysin (MRQ-40) and chromogranin (LK2H10) in the Merkel cell carcinoma, besides positive cytokeratin 20 (clone SP33) with a “dot” pattern ‒ these markers were negative in the squamous cell carcinoma. There was also negative TTF1 (clone 8G7G3/1), and positive cytokeratin 5/6 (D5/16B4) and p63 (clone 4A4) in the squamous cell carcinoma (Fig. 3). On physical examination, the patient showed no signs of lymph node enlargement and was referred to Oncology for clinical staging and treatment. The tumor polyomavirus status was not tested or reported.

**Fig. 1:**
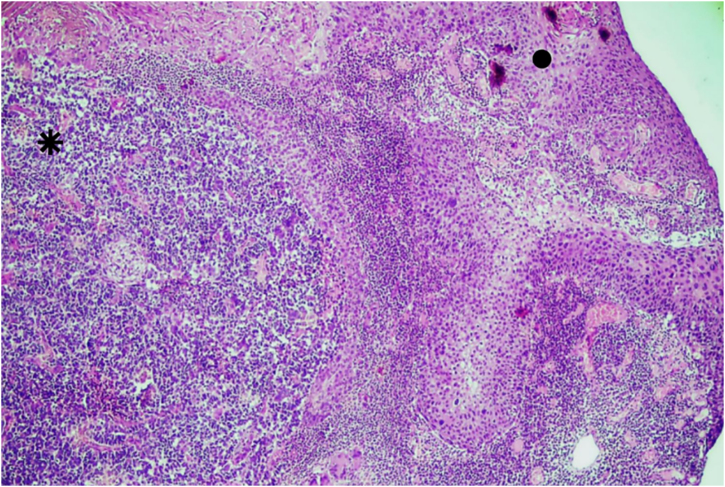
Squamous cell carcinoma (●) coexisting with Merkel cell carcinoma (⁕). Hematoxylin & eosin, ×40.

**Fig. 2:**
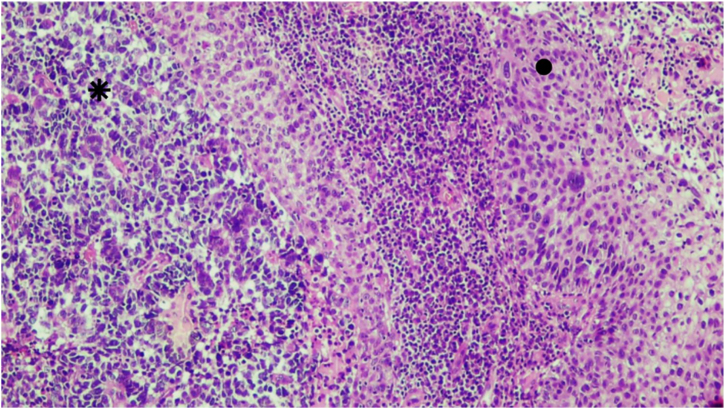
Squamous cell carcinoma (●) coexisting with Merkel cell carcinoma (⁕). Hematoxylin & eosin, ×100.

**Fig. 3:**
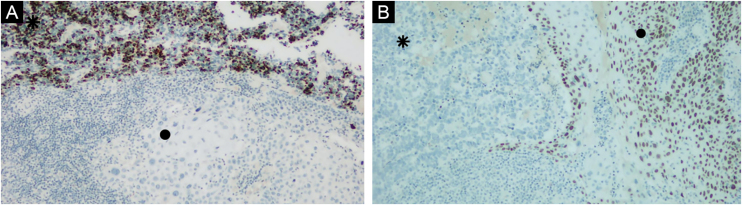
(A) CK20 positive “dot” pattern in Merkel cell carcinoma (⁕) and negative in squamous cell carcinoma (●). CK20 (SP33), ×100. (B) P63 negative in Merkel Cell Carcinoma (⁕) and positive in squamous cell carcinoma (●). P63 (4A4), ×100.

Merkel cell carcinoma, or primary neuroendocrine carcinoma of the skin, is a rare and aggressive malignant neoplasm. It was first reported by Toker in 1972 as “trabecular carcinoma”.^5^ In 2008, the polyomavirus associated with Merkel cell carcinoma was discovered, called Merkel cell polyomavirus (MCPyV).^6^ Clinically, its presentation usually consists of an asymptomatic plaque or nodule, pink or reddish-blue in color, sometimes ulcerated, and showing rapid growth. Histopathologically, it presents as an ill-defined dermal nodule that can infiltrate fatty tissue, fascia, and muscle. It is characterized by the monotonous proliferation of small, round to oval cells with basophilic nuclei and dispersed nuclear chromatin.^1^, ^3^, ^7^ It expresses neuroendocrine markers, including chromogranin, synaptophysin, neuron-specific enolase, and CD56 in immunohistochemical studies.^8^, ^9^ Another expressed marker – and the most specific one, with a “dot” pattern – is CK20.^1^ Staining for TTF1 is usually negative.^4^

MCC may occasionally be associated with other skin neoplasms. The most common association, although rare, is between MCC and *in situ* or invasive squamous cell carcinoma. The rarity of the lesion has prevented the correct quantification of the association, since most of the published data come from case reports.^3^ A recent multi-institutional study analyzed 136 MCCs and found a 10% frequency of MCC association with *in situ* or invasive SCC, compared with two other series in the literature: one in which this percentage was 10.34% and the other 6.25%. These studies describe all associations between MCC and SCC, which include (1) intraepidermal MCC within an *in situ* SCC, (2) MCC with *in situ* SCC, and (3) MCC associated with *in situ* and invasive SCC. Additionally, other studies report “mixed tumors” and divergent differentiation in MCC ‒ presence of squamous differentiation.^1^

The presence of polyomavirus has been widely studied in MCC. A study published in 2009 that investigated the presence of polyomavirus in MCC using various techniques showed that immunohistochemical testing using the monoclonal antibody CM2B4 proved valid, since all tumors immunoreactive with CM2B4 were positive in the polymerase chain reaction (PCR) technique. Also in this study, seven MCCs associated with SCC were evaluated and all were positive for CK20, but negative for CM2B4 (both in the neuroendocrine and squamous cell components).^10^ Associated with the fact that both neoplasms share common risk factors, such as sun exposure, low phototype and advanced age, it may be suggested that MCC associated with SCC may develop through a polyomavirus-independent pathway.

The present report describes a case of Merkel cell carcinoma associated with invasive squamous cell carcinoma. Despite its rarity, such an association, already reported in the literature, raises aspects about its histogenesis, which are still the subject of studies and discussion.

## Financial support

None declared.

## Authors’ contributions

Mariana Abdo de Almeida: Approval of the final version of the manuscript; design and planning of the study; drafting and editing of the manuscript; collection, analysis and interpretation of data; effective participation in research orientation; intellectual participation in the propaedeutic and/or therapeutic conduct of the studied cases; critical review of the literature; critical review of the manuscript.

Neusa Yuriko Sakai Valente: Approval of the final version of the manuscript; design and planning of the study; drafting and editing of the manuscript; collection, analysis and interpretation of data; effective participation in research orientation; intellectual participation in the propaedeutic and/or therapeutic conduct of the studied cases; critical review of the literature; critical review of the manuscript.

Mariangela Cristina Crispino Barata: Approval of the final version of the manuscript; design and planning of the study; drafting and editing of the manuscript; collection, analysis and interpretation of data; effective participation in research orientation; intellectual participation in the propaedeutic and/or therapeutic conduct of the studied cases; critical review of the literature; critical review of the manuscript.

Emílio Martins Curcelli: Approval of the final version of the manuscript; drafting and editing of the manuscript; collection, analysis and interpretation of data; intellectual participation in the propaedeutic and/or therapeutic conduct of the studied cases; critical review of the manuscript.

Bruna Nascimento Arruda Scabello: Approval of the final version of the manuscript; drafting and editing of the manuscript; collection, analysis and interpretation of data; intellectual participation in the propaedeutic and/or therapeutic conduct of the studied cases; critical review of the manuscript.

## Conflicts of interest

None declared.
